# Glioma stem cells enhance endothelial cell migration and proliferation via the Hedgehog pathway

**DOI:** 10.3892/ol.2013.1569

**Published:** 2013-09-09

**Authors:** GUANG-NING YAN, YANG-FAN LV, LANG YANG, XIAO-HONG YAO, YOU-HONG CUI, DE-YU GUO

**Affiliations:** Department of Pathology, Southwest Hospital, Third Military Medical University of PLA, Shapingba District, Chongqing 400038, P.R. China

**Keywords:** glioma stem cell, Hedgehog pathway, endothelial cell, migration, proliferation

## Abstract

The aim of the present study was to determine the possible mechanism underlying the enhanced migration and proliferation of endothelial cells caused by glioma stem cells (GSCs). Tumor spheres enriched in GSCs derived from the mouse GL261 glioma cell line, and the brain microvessel endothelial cell line, b.END3, were used in this study. A Transwell co-culture system, RNAi experiments, quantitative polymerase chain reaction, western blotting and enzyme-linked immunosorbent, cell counting kit-8 (CCK-8) proliferation, Transwell migration and wound-healing assays were used in this study to determine the migration and proliferation ability, as well as the Hedgehog (HH) pathway-related gene expression in the b.END3 cells. Based on the results, it was demonstrated that the migration and proliferation of the endothelial cells were enhanced following co-culture with GSCs. The gene expression of the HH pathway-related genes, Sonic Hedgehog (Shh) and Hedgehog-interacting protein (Hhip) was altered in the endothelial cells when co-cultured with GSCs. Overexpression of glioma-associated oncogene homolog 1 indicated activation of the HH pathway. Following knockdown of smoothened (Smo) in the endothelial cells, the migration and proliferation abilities of the cells were inhibited. GSCs have little effect on enhancing these behaviors in endothelial cells following Smo-knockdown. Further investigation revealed that Shh levels in the supernatant of the co-culture system were elevated, indicating the importance of secreted Shh from the endothelial cells. In conclusion, GSCs enhanced the migration and proliferation of the endothelial cells *in vitro*, which was likely associated with the activation of the HH pathway in the endothelial cells, caused by the increased secretion of Shh.

## Introduction

Glioblastoma multiforme (GBM) has been one of the most lethal malignant brain tumors in adults for the past 30 years. The best treatment to date is a combination of surgery, radiation therapy and temozolomide administration; however, this does not produce sufficient results. The overall survival time of patients with GBM is between 26 and 52 weeks (1–3). Previous studies have identified that a small population of tumor cells called glioma stem cells (GSCs) is responsible for GBM initiation, propagation, resistance and recurrence (4,5). Thus, GSCs have become one of the hot topics of glioma research.

Solid tumors are unable to grow without the help of vessels; necrosis appears in the center of a tumor when the volume of the tumor reaches 8 mm^3^ without new vessels (6). Angiogenesis is one of the most significant characteristics of malignant neoplasms, including endothelial cell migration, proliferation and vessel remodeling. Glioblastomas are rich in microvessels. Previous studies have demonstrated that GSCs co-localize with microvessels (7), have a positive correlation with microvessel density (MVD) (8) and have multiple regulatory roles in endothelial cells (9).

The Hedgehog (HH) pathway is extremely important in embryonic development, since it directs embryonic growth and cell fate determination. The canonical HH pathway is activated by HH ligands, mostly by Sonic Hedgehog (Shh) in the nervous system, which binds to receptor complexes, such as the transmembrane protein, Patched, and the G-protein-coupled protein, Smoothened (Smo). Hedgehog-interacting protein (Hhip) is one of the inhibitory ligands that binds to Shh. The binding of Shh to Patched releases Smo. Smo then alters the activity of glioma-associated oncogene homolog 1 (Gli1) protein and initiates the transcription of downstream genes. The HH pathway is expressed in endothelial cells. It has been demonstrated that the proliferation and migration of endothelial cells are associated with the HH pathway (10).

The aim of the present study was to investigate whether GSCs enhance proliferation and migration of endothelial cells, and whether the HH pathway plays a role in this process.

## Materials and methods

### Cell culture

The mouse GL261 glioma and brain microvascular endothelial cell lines (Cell Bank of Shanghai Institute of Cell Biology, Chinese Academy of Sciences, Shanghai, China) were used in this study. The two cell lines were cultured in Dulbecco's modified Eagle's medium (DMEM), containing 10% fetal bovine serum (FBS), 100 U/ml penicillin and 100 mg/ml streptomycin. GSC spheres were obtained from GL261 cells and cultured in serum-free DMEM/F12 medium with 10 ng/ml epidermal growth factor (EGF; PeproTech, Inc., Rocky Hill, NJ, USA), 10 ng/ml basic fibroblast growth factor (bFGF; PeproTech) and 2 mg/ml B27 (Sigma-Aldrich, St. Louis, MO, USA) (11,12).

For the co-culture system, Transwell cell culture inserts were used (0.4 μm; Millipore, Billerica, MA, USA) to explore the indirect effect of GSCs on endothelial cells. Briefly, 2×10^5^ b.END3 cells were seeded in 6-well plates, while 1.5×10^5^ GSCs were seeded in the cell culture chamber. The insert was transfered into the well with endothelial cells after 24 h of culture.

### Transwell migration assay

Transwell cell culture inserts were selected (8.0 μm; Millipore). The cells were trypsinized and counted, then 1.5×10^5^ cells were seeded in each cell culture insert. Serum-free cell culture medium (400 μl) was added to each insert. One milliliter medium with 5% FBS was added to the 6-well plate, then the insert was transferred to the well and cultured at 37°C. At the end of the migration assay, the insert was washed with phosphate-buffered saline (PBS) and fixed in 4% paraformaldehyde. The cells that were unable to migrate were wiped out and the rest were stained by crystal violet solution (Beyotime Institute of Biotechnology, Beijing, China). The cells were counted under a microscope at each field.

### Wound-healing assay

The b.END3 cells (2×10^4^) were seeded in a 6-well plate cultured with DMEM in serum. Pipette tips (200 μl) were used to scratch three parallel vertical lines in each well subsequent to 12 h of culture. The wells were washed with PBS, then the medium was changed to serum-free DMEM. Scratch lines were observed under a microscope and scratch distances were measured, with images captured at 6, 8, 12 and 24 h after scratching.

### Gene knockdown assay

The murine Smo mRNA sequence (NM_176996.4) was acquired from the NCBI database. Murine shRNA targeted to Smo was designed, synthesized and packaged with lentivirus by SBO Biomedical Technology (Shanghai, China). The endothelial cells were seeded in a 6-well plate at 1×10^5^ cells/well. Subsequent to 24 h of culture, the lentivirus was diluted and added to culture medium according to the manufacturer's instructions. The cells were purified following proliferation.

### Cell counting kit-8 (CCK-8) proliferation assay

A CCK-8 proliferation assay kit (Beyotime Institute of Biotechnology) was used in this experiment. A 48-h co-culture medium and a 48-h control well culture medium were collected from the empty control vector (NC b.END3; medium a and b) and siR-Smo b.END3 cells (medium c and d). NC b.END3 and siR-Smo b.END3 cells were seeded separately in a 96-well plate at 3×10^3^ cells/well, using 40 wells each. Co-cultured medium or control medium (both 150 μl) or 50 μl fresh medium was added to each well subsequent to 12 h of culture. The specific grouping was as follows: 40 wells with NC b.END3 plus medium a, 40 wells with NC b.END3 plus medium b, 40 wells with siR-Smo b.END3 plus medium c and 40 wells with siR-Smo b.END3 plus medium d. At each time-point, the medium in each well was changed to 200 μl fresh medium mixed with 10 μl CCK-8 solution for 10 wells from each group. The absorbance at 450 nm was detected following 2 h of incubation.

### Quantitative polymerase chain reaction (qPCR)

The total RNA of the b.END3 cells was extracted with RNAiso reagent (Takara Bio Inc., Shiga, Japan). Reverse transcription and PCR were performed using a Takara RNA PCR (AMV) kit with primers designed for murine genes. The primers were synthetized by Invitrogen (Carlsbad, CA, USA). The sequences of each primer pair and the product size are presented in Table I. The results were normalized against the level of glyceraldehyde 3-phosphate dehydrogenase (GAPDH) as the internal control. qPCR was performed in triplicate for each experiment, including for the non-template controls.

### Western blotting

The proteins of the b.END3 cells were extracted by RIPA lysis buffer (Beyotime Institute of Biotechnology) with the protease inhibitor phenylmethanesulfonyl fluoride (Beyotime Institute of Biotechnology). The proteins were separated with 10% sodium dodecyl sulfate-polyacrylamide gel electrophoresis (SDS-PAGE) and transferred onto polyvinylidene difluoride (PVDF) membranes (Millipore). The membranes were blocked with 5% milk, then incubated with primary rat anti-mouse Gli1 (1:1,000; R&D Systems, Minneapolis, MN, USA), rat anti-mouse Shh (1:1,000; R&D Systems), rabbit anti-mouse Hhip (1:1,000; Abcam, Cambridge, MA, USA) and rabbit anti-mouse GAPDH (1:1,500; Goodhere, Hangzhou, China) antibodies overnight at 4°C. The membranes were then washed and incubated with goat anti-rat and goat anti-rabbit horseradish peroxidase (HRP)-conjugated secondary antibodies (1:100; ZSGB-BIO, Beijing, China) for 2 h at room temperature. The proteins were detected by enhanced chemiluminescence detection reagent. GADPH was used as the loading control.

### Enzyme-linked immunosorbent assay (ELISA)

Cell culture supernatant was acquired from the wells of the GSCs, b.END3 cells and the co-culture. Medium was removed and centrifuged at 3,000 × g after 12, 24 and 48 h of culture, then stored at −80°C immediately. An ELISA kit for mouse Shh N-Terminus was purchased from R&D Systems and an ELISA kit for mouse Hhip was purchased from USCN Life Science Inc. (Wuhan, China). ELISA was performed strictly according to the manufacturer's instructions.

### Statistical analysis

All experiments were conducted at least three times and the results were from representative experiments. Data are expressed as the mean ± standard deviation and the statistical significance between the experimental and control groups was analyzed with SPSS 16.0 statistical software (SPSS Inc., Chicago, IL, USA). When two groups were compared, the unpaired Student's t-test was used. P<0.05 was considered to indicate a statistically significant difference.

## Results

### Migration of endothelial cells is enhanced when cultured with GSCs

**Figure 1:**
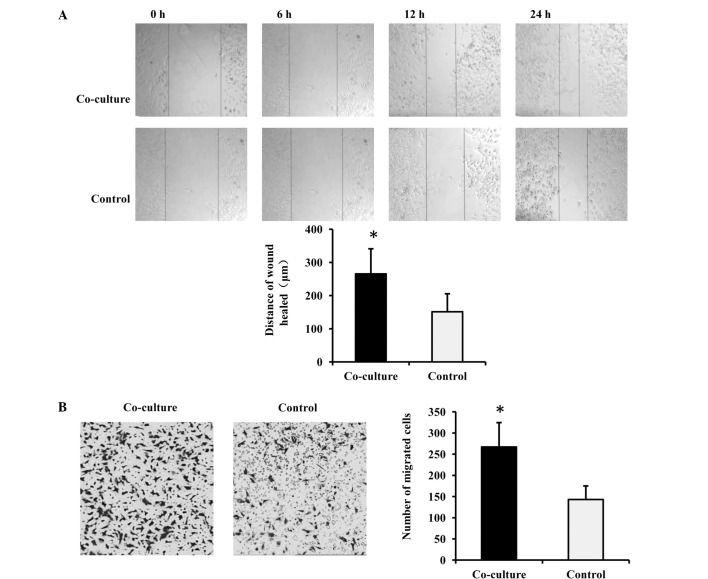
Migration of endothelial cells enhanced by glioma stem cells (GSCs). (A) Endothelial cells in the wound-healing assay migrated faster when cultured with GSCs compared with the control. (B) The endothelial cells had an enhanced migration ability under serum chemotaxis following culture with GSCs, compared with the control cells. ^*^P<0.05 vs. control.

To explore the effect of GSCs on endothelial cells, a Transwell co-culture system was selected for the *in vitro* model in order to rebuild an approximate *in vivo* niche, which we consider to be better than just using a GSC-conditioned medium. In this model, the two types of cells interact via soluble factors, but do not have direct connections. The b.END3 cells were seeded in the lower chambers and GSCs were seeded in the upper chambers. When the wound-healing assay was processed in the co-culture wells, it was clear that the co-cultured b.END3 cells exhibited enhanced migration, since the scratches in the co-cultured wells were narrower than in the control (Fig. 1A). The endothelial cells in tumor angiogenesis were guided by chemokines, so a Transwell migration assay was generated to confirm the observation with the help of serum. Subsequent to co-culture for 48 h, more b.END3 cells migrated through the membrane and appeared on the other surface (Fig. 1B). This result indicated that GSCs enhanced the migration of the endothelial cells.

### Proliferation of endothelial cells is enhanced by GSCs

**Figure 2:**
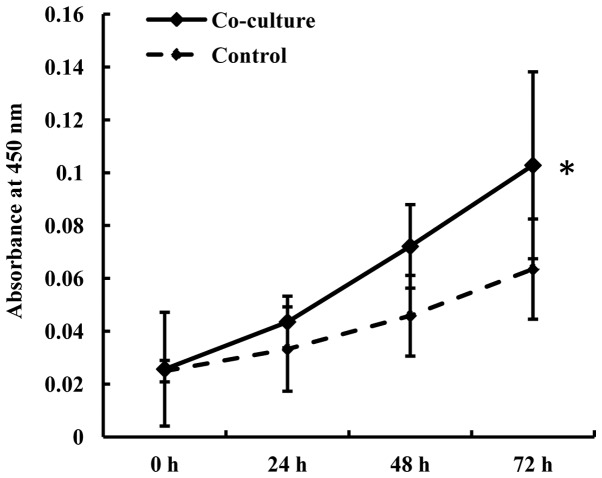
Proliferation of b.END3 cells enhanced by glioma stem cells. ^*^P<0.05 vs. control.

A proliferation assay was performed to determine whether GSCs would affect the proliferation ability of the endothelial cells. The endothelial cells were cultured with or without GSCs for 48 h, then seeded in a 96-well plate. The medium in each well consisted of 50 μl fresh medium mixed with 150 μl 48-h co-cultured medium or 150 μl control medium, respectively. The proliferation of the b.END3 cells was shown to be accelerated after co-culture and was positively related to the culture time (Fig. 2).

### HH pathway in endothelial cells is activated by GSCs

**Figure 3:**
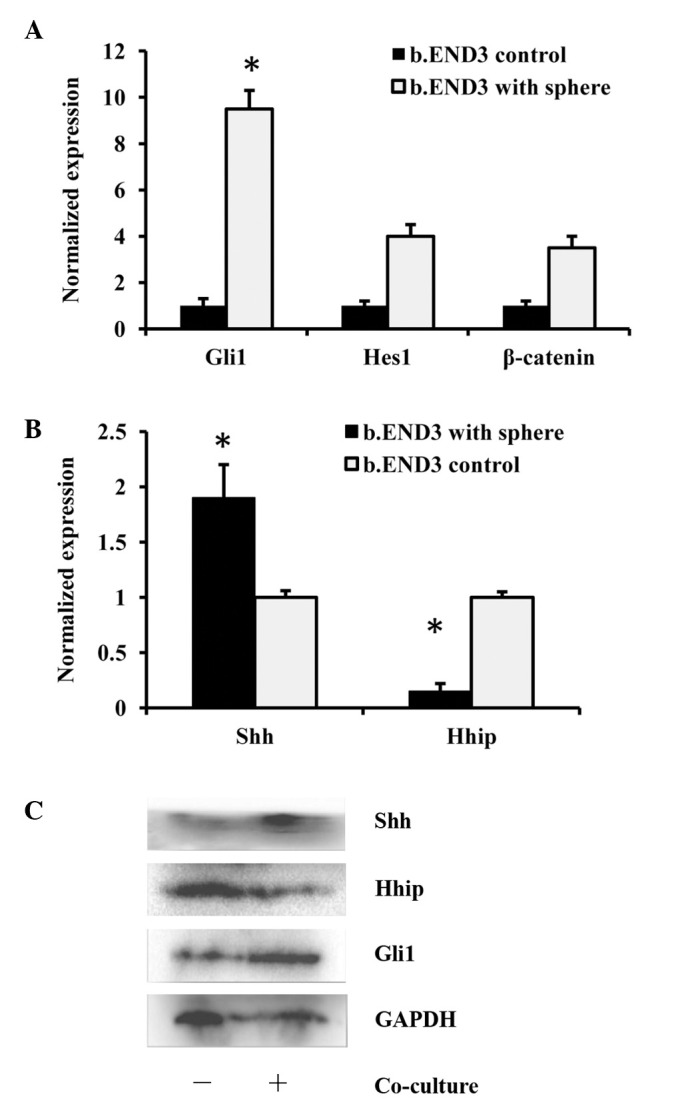
Activated Hedgehog (HH) pathway in the endothelial cells when cultured with glioma stem cells (GSCs). (A) Compared with Hes1 and β-catenin, glioma-associated oncogene homolog 1 (Gli1) was significantly upregulated in the b.END3 cells at the mRNA level. (B) Sonic Hedgehog (Shh) was upregulated and Hedgehog-interacting protein (Hhip) was downregulated in the b.END3 cells at the mRNA level when cultured with GSCs. (C) Western blotting confirmed these expression changes. ^*^P<0.05 vs. control. GAPDH, glyceraldehyde 3-phosphate dehydrogenase.

To determine the mechanism behind the migration and proliferation of the endothelial cells caused by GSCs, three possible pathways were selected that may have been involved. The HH, Notch and β-catenin pathways all participate in endothelial cell proliferation, migration, angiogenesis and the functioning of endothelial cells. Although the three pathways were all affected in the b.END3 cells following the 48-h co-culture with GSCs, the Gli1 gene, which is the key component of the HH pathway, was induced to the highest extent at the mRNA level (Fig. 3A). It was also demonstrated that ligands of the HH pathway, Shh and Hhip, had altered expression (Fig. 3B), which was confirmed at the protein level (Fig. 3C). These results indicated that the HH pathway may be the main mediator of the effect of GSCs on the b.END3 cells.

### Migration ability of endothelial cells is inhibited following Smo gene knockdown

**Figure 4:**
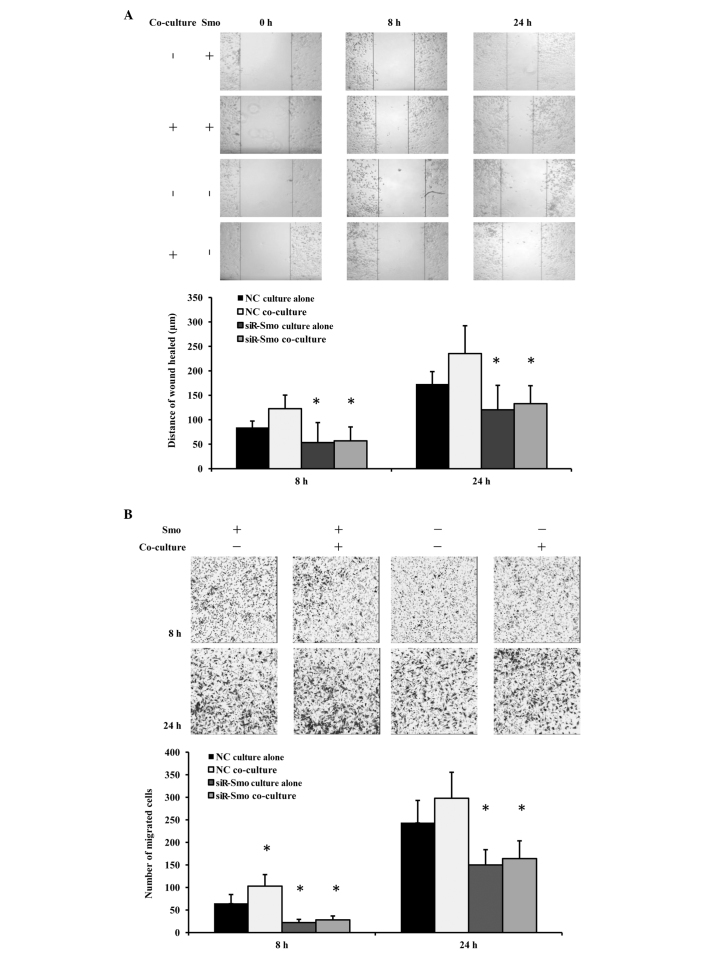
Reduced migration of the endothelial cells after Smoothened (Smo) gene knockdown. (A) Wound-healing ability was inhibited when the Smo gene was knocked down in the b.END3 cells. (B) Migration ability was inhibited when the Smo gene was knocked down in the endothelial cells. ^*^P<0.05 vs. corresponding control. NC, empty control vector; siR-Smo, Smo knockdown.

To further confirm the interaction of the HH pathway in GSC-enhanced b.END3 cell mobility, Smo gene expression was knocked down in the b.END3 cells, then the HH pathway was partially blocked. Migration assays were repeated using siR-Smo-b.END3 cells and control cells. Early and late time-points were selected in order to observe the effect pattern. As expected, the migration ability of the b.END3 cells was inhibited when the HH pathway was knocked down. Furthermore, the siR-Smo-b.END3 cells co-cultured with GSCs did not retrieve the normal level of migration ability in the wound-healing assay (Fig. 4A) and Transwell migration assay (Fig. 4B). These results indicated that the HH pathway was the molecular mechanism behind the effect of GSCs on the migration ability of the b.END3 cells. Additionally, it was observed that the phenomenon was more significant at an early stage than a late stage. We suspect that this was due to the unstable overactivation of the HH pathway.

### Proliferation of endothelial cells is reduced following Smo gene knockdown

**Figure 5:**
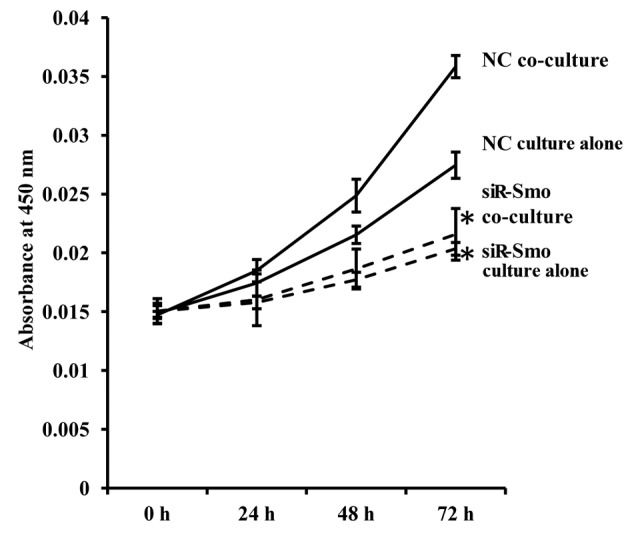
Reduced proliferation of the endothelial cells following Smoothened (Smo) gene knockdown. NC, empty control vector; siR-Smo, Smo knockdown. ^*^P<0.05 vs. corresponding control.

The proliferation assay was repeated to explore the importance of the HH pathway in GSC-enhanced b.END3 cell proliferation. Consistent with our expectations, the proliferation of the endothelial cells was significantly decreased after Smo was inhibited, and this was not restored by culture with GSCs (Fig. 5).

### Shh and Hhip secretion is altered in the co-culture system

**Figure 6:**
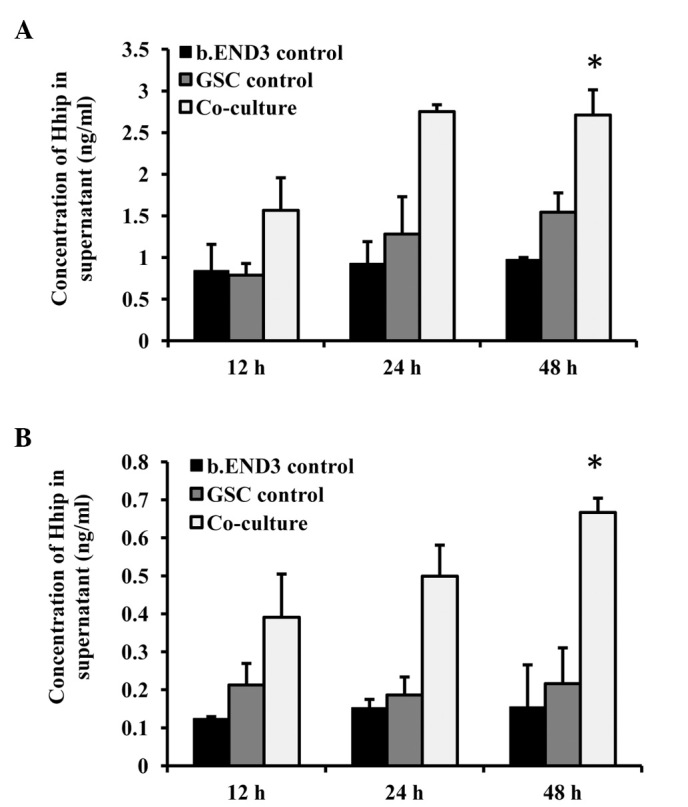
Hedgehog (HH) ligand expression was altered in the co-culture system. (A) Higher levels of Sonic Hedgehog (Shh) were detected in the supernatant of the co-culture wells compared with the b.END3 control cells and the glioma stem cell (GSC) control wells. (B) Higher levels of Hedgehog-interacting protein (Hhip) were detected in the supernatant of the co-culture wells compared with the b.END3 control cells and the GSC control wells. ^*^P<0.05 vs. control.

To further explore the upstream factor of the HH pathway in the co-culture, Shh and Hhip, the activator and inhibitor of the HH pathway, were detected. As we considered that the regulation of the endothelial cells by the GSCs *in vivo* was mediated indirectly due to the low percentage of GSCs, ELISA was used to detect the amount of Shh and Hhip secreted into the supernatant. The Shh concentration was increased in the co-cultured wells dependent on the culture time (Fig. 6A). A higher concentration of Hhip protein was also detected in the co-culture supernatant (Fig. 6B). Since Hhip was inhibitory, we hypothesize that the GSCs induced Shh expression, increasing the local concentration of Shh, and that Hhip expression was the feedback effect.

## Discussion

Glioma is the most common malignant tumor in the brain, accounting for 33.3–58.9% of all brain tumors, and with an incidence that is still increasing (13). Modern surgery and other treatments are not effective enough, due to the unique biological behavior, high invasive growth and recurrence of glioblastomas. GSCs have been isolated and identified. Researchers hope this small group of cells, which self-renew and undergo multipotential differentiation, are responsible for glioblastoma initiation, propagation and recurrence, and may provide an indication towards a cure (4,14).

Angiogenesis is closely related to tumor growth. GBM is rich in microvessels, making it a perfect model to study angiogenesis and cancer stem cells (CSCs). It has been discovered that the location of GSCs is close to the microvessels (10). Tumor microvessel endothelial cells have been shown to be morphologically different from normal endothelial cells, with elevated migration and resistance to necrosis (15). The mechanism under tumor angiogenesis is complex. Angiogenesis begins with the gemmation and migration of endothelial cells. Cell cords then form by endothelial cell proliferation and circulating endothelial cell recruitment. Microvessels mature with a series of vascular remodeling.

The mechanism of GSC-regulated tumor angiogenesis is a hot topic of research (16–18). Vascular endothelial growth factor is considered to be one of the most important factors (19). Bone morphogenic protein (20), formyl peptide receptor (21), stromal cell derived factor 1 and its receptor CXCR4 (22) and hypoxia-inducible factor (23) have all been reported to participate in the regulation of angiogenesis.

The present study indicates a new mechanism of tumor angiogenesis. The results demonstrated that GSCs regulate the gene expression of nearby endothelial cells, specifically through the HH pathway, thereby affecting their biological behavior. The HH pathway is a classical pathway of cell survival, proliferation and migration, and Shh is one of its classical activators, which functions in autocrine and paracrine ways (24). The HH pathway is activated in endothelial cells and their progenitors. Overexpression of the HH pathway affects the proliferation, migration and remodeling of vasculature (25). In the present study, it was observed that the Gli1 gene, which is the key gene in the HH pathway, was overexpressed in the endothelial cells when indirectly co-cultured with GSCs, indicating HH pathway activation. The proliferation and migration of the endothelial cells were induced. Once the HH pathway had been knocked down, the phenomenon disappeared. Next, the cause of this regulation was investigated and Shh was shown to be overexpressed and secreted in the endothelial cells when cultured with GSCs. Therefore, GSCs may regulate Shh expression in nearby endothelial cells via soluble factors, then activate their HH pathway. The present results also provide further evidence of a CSC niche where GSCs interact with endothelial cells.

Progression has been made in the study and treatment of cancer using anti-angiogenesis drugs. However, adverse effects have been observed. Microvasculature fracture causes local anoxia, which is another niche for CSCs, causing local appearance of the cells. In future studies, we aim to interfere with the interaction between GSCs and microvessels, for example the HH pathway, to inhibit tumor invasion and propagation and to provide opportunities for tumor resection.

## Figures and Tables

**Table I:** Primers used in this study.

Gene	Primer sequence (5′→3′)	Product size, bp
GAPDH	F: CCTGCACCACCACATGCTTA	85
	R: TCATGAGCCCTTCCACAATG	
Gli1	F: GAGAATGGGGCATCGTCGTCA	168
	R: CGGGTACTCGGTTCGGCT	
Hes1	F: TCAACACGACACCGGACAAAC	155
	R: ATGCCGGGAGCTATCTTTCTT	
β-catenin	F: GCTTCTATGAAGACCCCAGTTC	311
	R: CAGTGGGCTAGGTGTCAGGA	
Shh	F: AGGGGGTTTGGAAAGAGG	168
	R: GTCGGGGTTGTAATTGGG	
Hhip	F: GAGAAGGGACAGGCGGGTGA	212
	R: GGGAATGCGGGGAGCAGGGA	

F, forward; R, reverse; GAPDH, glyceraldehyde 3-phosphate dehydrogenase; Gli1, glioma-associated oncogene homolog 1; Shh, Sonic Hedgehog; Hhip, Hedgehog-interacting protein.
